# Nurse Leaders' Motivational Forces in Developing a Health-Promoting Work Environment: A Hermeneutic Study

**DOI:** 10.1155/jonm/3040594

**Published:** 2024-12-30

**Authors:** Diako Morvati, Rita Solbakken, Jonas Rennemo Vaag, Yvonne Hilli

**Affiliations:** ^1^Faculty of Nursing and Health Sciences, Nord University, Bodø, Norway; ^2^Department of Psychology, Inland School of Business and Social Sciences, University of Inland Norway, Lillehammer, Norway

**Keywords:** caring science, hermeneutic, motivation, nursing leadership/management, workplace

## Abstract

**Background:** The literature review highlights that a motivated leader plays a key role in motivating employees and fostering a health-promoting work environment. Despite its importance, there is limited knowledge about the motivational forces, from a caring science perspective, that drive nurse leaders to develop a health-promoting work environment.

**Aim:** This study aims to gain a deeper understanding of the motivational forces driving nurse leaders toward developing a health-promoting work environment.

**Method:** A qualitative design with a hermeneutic approach inspired by Gadamer was chosen. Data were collected through semistructured interviews with 13 nurse leaders in northern Norway between December 2023 and February 2024. The Standards for Reporting Qualitative Research (SRQR) were followed in reporting the results.

**Findings:** The nurse leader's motivational forces in developing a health-promoting work environment imbued with an ethos of (1) sense of inner responsibility for promoting the wellbeing of others, (2) sense of trusting relationships in the workplace, (3) sense of mastery through learning and development, and (4) sense of recognition from superiors through being seen, supported, and involved.

**Conclusion:** This study highlights the importance of raising awareness and articulating nurse leaders' underlying motives and values. Doing so transforms abstract concepts into tangible factors that stakeholders can grasp. In particular, this insight provides valuable guidance to politicians and decision-makers on how to facilitate improved working conditions for nurse leaders, thereby maintaining and enhancing their motivation.

**Implications for Nursing Leadership:** The findings of this study recommend that superior leaders and policymakers should prioritize involving nurse leaders in decision-making and promoting their autonomy and room for action. In addition, it suggests facilitating further education and training programs for leaders, as well as maintaining leadership networks where nurse leaders can reflect, exchange experiences, and address challenges.

## 1. Introduction/Background

Nurse leaders play a key role in developing a health-promoting work environment [1–5]. A health-promoting work environment is crucial in nurses' job satisfaction, reducing turnover, stress, and burnout while fostering higher nurse retention rates [1, 2, 6–8]. Moreover, it improves overall practice, enhances the quality of care, and ensures patient safety in the healthcare setting [9, 10]. In this study, “nurse leader” refers to individuals with a nursing background in a formal mid-level leadership position, responsible for personnel, finances, and patient care in clinical practice.

The Norwegian healthcare system is founded on the principles of universal access; it is a government-controlled healthcare system financed by taxation [11]. Like other countries, the Norwegian healthcare service has undergone reforms to align with new public management since the 1990s. This management ideology imposes efficiency and productivity demands on leadership, emphasizing skills in a more market-oriented direction [12, 13]. According to previous studies, being a nurse leader in such a context means living in two different worlds, which implies a tension between various values, that is, the relationship between inner responsibility—responsibility for our fellow human beings—and outer responsibility—tied to external demands for financial and administrative duties [14–16].

Characteristics of various forms of value-based leadership, including servant leadership [17], charismatic leadership [18], transformational leadership [19], and authentic leadership [20, 21] represent leadership styles that foster health, job satisfaction, and employee engagement. Authentic leadership has been described as the “glue” needed to hold together a healthy work environment [4, 22]. Authentic leadership is defined by four constructs: self-awareness (reflecting on one's own core values, goals, and motives), internalized moral perspective (practices guided by core personal values and moral standards), relational transparency (being open and honest to others), and balanced processing (an individual's ability to analyze information objectively and explore other people's opinion before making decisions) [23, 24]. The consciousness of one's values and motives is the essence of authentic leadership [23, 25], and there is a positive relationship between authentic leadership and high levels of motivation [26]. According to Zhang and Fan [27], motivated leaders demonstrate responsibility, initiative, and a positive attitude, along with a willingness to cultivate a work environment that promotes health for their employees. Motivation thus plays a crucial role in nursing leadership, driving efforts to attain organizational goals, gain valued outcomes such as improved patient outcomes and quality of nursing care standards, as well as shaping behavior and guiding decision-making processes [28, 29].

The understanding of motivation has developed throughout history. Previously, the emphasis was placed on incentives or extrinsic motivation, specifically rewards and punishments, as significant elements in driving performance motivation [30]. However, in recent years, the attention has shifted toward identifying factors enhancing intrinsic motivation. According to Deci and Ryan's self-determination theory, human beings have three essential psychological needs: autonomy, competence, and relatedness. They argue that intrinsic motivation emerges when these foundational psychological needs find fulfillment. In activities driven by intrinsic motivation, the inherent satisfaction and enjoyment derived from the experience itself act as the main “reward.” Conversely, externally motivated behavior involves participating in an activity because someone else decides it or because we want to achieve something other than the joy of the activity itself. In other words, extrinsic motivation encompasses all instrumental behaviors [31, 32]. In this study, external factors such as salary and status are understood in accordance with Herzberg's theory. This theory implies that these hygiene factors can lead to dissatisfaction if absent; however, their presence does not necessarily result in wellbeing and motivation [33].

From a caring science perspective on which this study is based, motivation is understood as the driving force that compels a person to act based on core values or ethos, which implies showing respect for human dignity and integrity, having the courage to take on responsibility and being engaged [34, 35]. The source of motives is not merely external but lies deep within an individual, metaphorically described as the innermost room of the human being. In this innermost room, individuals discover their ethos that fosters a good life where a person metaphorically feels “at home.” The home as the ethos comprises three dimensions. The innermost dimension is imbued with a person's basic values and ethos. The middle dimension encompasses a person's manner of being and ethical code of conduct, all shaped by the ethos and reflected in the third, physical, and outermost dimensions, where persons meet, act, and interact [34]. The ethos of caring in nursing leadership arises from an inner motivational force grounded in a love of humanity and mercy, aiming to promote health, alleviate suffering, and respect human dignity [36, 37]. Caring in leadership also stems from an inner motive to take responsibility and act in the best interests of patients. Caring for staff and caring for patients were described as two sides of the same coin. When leaders care about their staff, they also care for their patients [38]. Leaders need to be aware of their basic values and ensure that these values are reflected in their actions, interactions in the workplace, and the exercise of their leadership. This is essential if they are to be seen as authentic and caring [16, 36]. In previous studies, leaders have highlighted that their basic values of responsibility, respect, and recognition of the uniqueness of patients and employees serve as an inner driving force to be engaged leaders in promoting a caring culture [16, 38, 39].

The literature review highlights that a motivated leader plays a vital role in motivating employees and fostering a health-promoting work environment. Despite its importance, there is limited knowledge about the motivational forces, from a caring sciences perspective, that drive nurse leaders to develop a health-promoting work environment. This study aims to address this knowledge gap from the viewpoints of nurse leaders.

## 2. Aim

This study aims to gain a deeper understanding of the motivational forces driving nurse leaders toward developing a health-promoting work environment.

## 3. Method

### 3.1. Research Design

This study used a qualitative design with a philosophical hermeneutic approach inspired by Gadamer [40]. According to Gadamer, understanding characterizes human beings, and all understanding depends on one's preunderstanding. Gadamer [40] emphasizes that understanding is achieved through a dialectical movement between the parts and the whole, the so-called hermeneutic circle or spiral. Openness is crucial for understanding, which can be demonstrated by asking questions and remaining open to the possibility that the answers may differ from expectations. One's understanding can thus be enriched through an open dialog with the participants, as well as with the transcribed text, which Gadamer calls “other horizons.” Other horizons refer to the perspectives, experiences, and various factors that, in this case, shape nurse leaders' understanding of what motivates them to develop a health-promoting work environment. When two horizons meet and a shared understanding emerges, the two horizons merge in what Gadamer calls a fusion of horizons, resulting in a new and common understanding [40].

### 3.2. Participants and Contexts

Thirteen nurse leaders in various contexts in both hospitals and municipal health services in northern Norway participated in this study. A purposive sampling strategy was chosen, with inclusion criteria requiring participants to (i) be a nurse leader in a formal midlevel leadership position, (ii) possess a minimum of a bachelor's degree in nursing, and (iii) work full-time (100%) as a leader, considering that some nursing leaders have reduced or shared positions. The rationale for selecting varied contexts and leaders with diverse experience backgrounds was to address the nuances and insights crucial for understanding the diversity in nursing leaders' motivational forces to develop a health-promoting work environment. Participation comprised 11 women and 2 men. The age range was 32–57 years. Two participants had 6 months of work experience as leaders, while the rest (*n* = 11) had more than 2 years of experience as leaders. Due to the limited participation of only two men in the study, along with the small number of hospitals and the small size of municipalities in northern Norway, characteristics are presented with caution to ensure anonymity (Table 1).

### 3.3. Data Collection

Qualitative semistructured, in-depth interviews were chosen as the data collection method. In studies inspired by philosophical hermeneutics, understanding emerges through dialog, with the researcher open to others' opinions and meanings. Consequently, engaging in conversations between the researcher and the participants is an appropriate method for grasping a phenomenon [41]. To increase awareness of preunderstanding, an interview guide was used. Data were collected between December 2023 and February 2024. The interviews were conducted in Norwegian and lasted between 45 and 60 min each. Six interviews were conducted via the digital platform Teams, and the rest were conducted at the nurse leaders' workplaces during their working hours. The interviews were recorded using audio recorders and transcribed verbatim immediately after each interview. After conducting 11 interviews, the data seemed to have reached a saturation point regarding meaningful content. Despite this, two additional interviews were conducted as a precaution to ensure that no new insights or themes emerged. This was done to ensure that the saturation point had been reached and that further interviews would not contribute new insights or understanding of the research question [42, 43].

### 3.4. Data Analysis

The data were interpreted using Gadamer's hermeneutic philosophy, following a four-step structure developed by Fleming et al. [41], which is grounded in Gadamer's hermeneutic philosophy (Table 2). This method was chosen to provide a structured framework that facilitates the interpretation process. The first author transcribed and analyzed the interviews, in Norwegian, engaging in frequent discussions and collaboration with other authors. Following several rounds of discussion, consensus was reached on the analysis and findings. The interpretation process, which was a dialectical movement between parts and the whole, started already during the interviews. The data were analyzed manually without the use of qualitative data analysis software, as we, based on our experience, find that a hermeneutic approach better preserves the holistic understanding of the text when employing a manual, pen-and-paper approach.

In the first phase, to gain a deeper and holistic understanding of leaders' experiences, the audio recordings and transcribed data were listened to and read several times. In the second phase, each sentence and passage was closely examined and interpreted to uncover in-depth nuances and meaningful units. In the third phase, the identified meaning units were related to the whole text. This process helped to establish connections and place the leaders' experiences in a broader context, enabling us to gain a more holistic understanding of leaders' motivational forces. Finally, in the fourth phase, we synthesized the leaders' experiences and our understanding of them to formulate main themes that could express a shared understanding between us as researchers and the participants. Through this process, our horizons merged with the participants' horizons, and a fusion of horizons emerged, presented as the study's findings.

### 3.5. Ethical Considerations

This study was approved by the Norwegian Agency for Shared Services in Education and Research (SIKT-Ref. no. 389393). All participants were provided with both written and oral information about the study. They were briefed on data privacy and informed of their right to withdraw from the study at any point. Before the interviews, participants confirmed their participation by signing a consent form. All participants' identifiable information was anonymized, and the audio files were deleted immediately after transcription.

## 4. Findings

**Figure 1:**
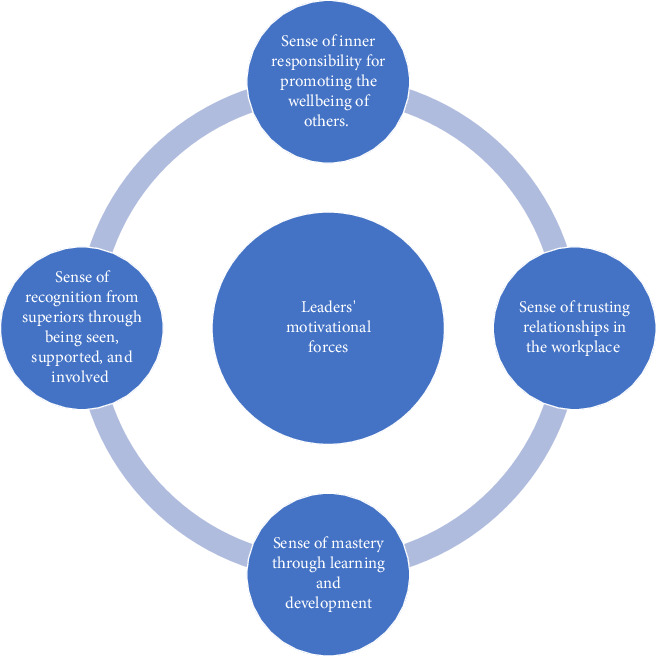
Nurse leaders' motivational forces in developing a health-promoting work environment.

The nurse leader's motivational forces in developing a health-promoting work environment imbued with an ethos of (1) sense of inner responsibility for promoting the wellbeing of others, (2) sense of trusting relationships in the workplace, (3) sense of mastery through learning and development, and (4) sense of recognition from superiors through being seen, supported, and involved (Figure 1).

### 4.1. Sense of Inner Responsibility for Promoting the Wellbeing of Others

Nurse leaders' sense of inner responsibility for promoting the wellbeing of other human beings is recognized as the fundamental motivational force driving them to develop a health-promoting work environment:

I've always had a great sense of responsibility. I've been very enthusiastic about taking responsibility… I've had a drive in me, that I've enjoyed helping others to become a better version of themselves. (P5)

While leaders' responsibility is primarily focused on patient care, they also emphasize the importance of fostering a work environment that prioritizes employees' needs and wellbeing in order to enhance their ability to provide high-quality care to patients:

I want the patients and their relatives to be as well as possible, and it's my job to make sure that the employees are both equipped and have the resources to do their job as well as possible. (P3)

This responsibility to create an environment where both patients and employees can thrive is deeply meaningful to these leaders. They derive deeper meaning and motivation from knowing they can make a positive difference in others' everyday lives:

What highly motivates me is the impact you have… the fact that you can make a difference. You can do something for others. (P4)

These insights imply that nurse leaders are driven by an altruistic attitude, indicating genuine concern for the wellbeing of others. Their sense of responsibility further emphasizes their commitment, motivation, and joy in helping and providing something valuable to others and the community. They consider this to be more meaningful than external factors, such as salary:

If you don't experience meaning at work, and if you only work for the money, you won't last. It's fine for two months, but then you get burnt out because you don't enjoy your job. (P7)

While external factors like salary may provide short-term motivation, the lack of meaningful work in the long term can demotivate leaders from cultivating a health-promoting work environment. This highlights the significance of interpersonal relationships as vital motivators in establishing meaningful working conditions, while also fostering engagement and wellbeing among both leaders and employees.

### 4.2. Sense of Trusting Relationships in the Workplace

As the leaders emphasized the importance of interpersonal relationships, they further highlighted trust as a crucial element in fostering these relationships within the work environment, which, in turn, is essential for maintaining their motivation.

Trusting relationships encompass three dimensions: trust between leaders and employees, between leaders and other leaders in midlevel positions, and between leaders and superior leaders. Trusting relationships between leaders and employees involve earning the employees' trust, so they feel comfortable seeking advice, giving feedback, and sharing their thoughts and concerns with their leaders:

It gives me a sense of motivation when people take the time to come in and talk to me. There must be a reason why they want to share their thoughts or things with me. (P8)

The leaders appreciate the trust extended to them, as it provides motivation and a sense of meaning to be someone others can trust. The leader–employee relationship also involves leaders trusting their employees and having confidence that they will carry out their tasks to provide high-quality care to patients:

I do not need to check up on them or anything like that, but I want them to be open and honest with me… It's very important to me that both they and I have that trust because I have to be able to trust them to do the right thing. (P3)

In addition to their relationships with employees, leaders emphasized the importance of building trusting relationships with other leaders in midlevel positions. They noted that leadership can be highly demanding, with constant pressures and expectations from various sources. Consequently, they view establishing trust with other leaders in similar positions as crucial for creating a space where they can share and reflect on their challenges:

There are some days that are demanding, so it's important that you have a leadership network around you that faces the same challenges… and there is room to be honest and talk about things that are difficult. (P11)

Furthermore, the trust between leaders and their superiors is expressed through the degree of discretion and freedom that leaders receive from their superior leaders. This reflects confidence in their abilities and competence, as granting them appropriate autonomy signals trust in their judgment and decision-making skills:

What kills the motivation is when I don't get the room for action, I need… I must be allowed to solve the tasks in my way. (P3)

It seems that having sufficient space for action and autonomy is crucial for leaders, as it helps them develop a sense of trust with their superiors. This trust is essential for enhancing the leader's motivation, as it allows them to take responsibility and actively contribute to fostering a healthy work environment. Conversely, restrictions on autonomy or a lack of trust can lead to a decline in motivation:

For me, it's very motivating to know that my leader trusts me…that she trusts me to do the tasks I'm supposed to do. I don't need micro-management … that someone can follow me in what I do. That's perhaps the most demotivating thing I know. (P5)

Leadership discretion, in turn, requires that leaders, especially those with less experience, acquire mastery through the provision of resources and training:

Yes… room for action is very important to me… but if I don't have the resources and information, I need to act within it… then I feel insufficient. (P12)

### 4.3. Sense of Mastery Through Learning and Development

As the leaders highlighted the importance of a sense of mastery, they emphasized that this sense is closely linked to their capability and ability to fulfill responsibilities toward individuals and the organization as a whole, aiming to improve the quality of care services:

What drives me is perhaps when we achieve what I really want to achieve. I have the regular tasks that need to be done, which are in my work instructions. (P1)

According to the leaders, achieving mastery requires continuous learning and professional development. Learning can either be in the form of facilitation and opportunities for further education and courses:

I was really inspired to …, I felt that it was the education (further education in leadership) where I both learned and practiced at the same time. (P3)

Or through the leadership training program especially for those with less leadership experience:

I want training to handle technical things, but I also want training for … it might be the employees that come with questions and things that I'm not sure how to solve (P7).

According to the leaders learning and training encompass administrative and technical aspects, addressing various interpersonal and ethical challenges that arise in everyday work. The leaders view learning as an ongoing process that enhances their competence by acquiring new skills and knowledge, leading to a sense of mastery over time. Therefore, personal development and mastery through learning can serve as motivators in cultivating a health-promoting work environment:

I get motivated when I experience personal development … Being able to see that I'm actually changing all the way … not only professionally, but also on a personal level (P11).

Furthermore, the leaders emphasize a dialectical and dynamic relationship between employee engagement and their own sense of mastery. When employees demonstrate engagement and handle situations independently, it provides leaders with a sense of mastery as well:

What is motivating for me is when they (employees) are concerned about professional development. (P7)

The leaders also expressed that when they feel they have acquired essential knowledge in developing a health-promoting work environment, it is important for their competence to be recognized:

It's if they (superior leader) say no… or decide that I shouldn't go ahead with the project that I've started,… it can actually make me unmotivated. (P13).

### 4.4. Sense of Recognition From Superiors Through Being Seen, Supported, and Involved

According to the leaders, they feel recognized when their superior leaders show care, provide feedback, and involve them early in the decision-making process. For the participants, experiencing care and support from their leaders was essential, particularly in having their efforts and contributions recognized:

Asking how things are going? …Being seen and recognized for the things you do… very simple things like being a fellow human being. (P1)

Being recognized as a fellow human being can be interpreted as being valued as a unique person with unique skills, qualities, and potential. This recognition serves as a source of motivation for leaders:

So it is simply very motivating that my leader takes the time to see me and the baggage that I have, and focuses on cultivating my positive qualities, but at the same time also made me aware of what I need to work on (P11)

The leaders emphasized that receiving feedback is a meaningful way to gain a sense of recognition:

It's very important that she (superior leader) gives me feedback and such. You become even more motivated to do a good job when you get feedback on what you do. (P9)

According to the leaders, recognition is also demonstrated through involvement in the decision-making process and having an impact on the final decisions. Some leaders noted that they are responsible for implementing these decisions in practice once they are made. Therefore, being involved early in the process encourages them to be more actively engaged in creating a health-promoting work environment:

I would have been involved in the decision-making process early enough. I may feel today that you're the last to know what's going to happen in the workplace… it's my responsibility… so it's very important to be involved and be able to influence the outcome. (P3)

When leaders are involved in the decision-making process, they also have the opportunity to express their opinions and make suggestions, which contributes to a sense of being seen and heard:

That you are involved. … that we have the opportunity to give our opinions and provide suggestions. If there are things that we think he needs to take further up the system. (P4)

However, when leaders experience a lack of recognition or feel that their voices are not heard by higher-level leadership, it can lead to frustration. This frustration can result in a feeling of working against their values, which would otherwise contribute to their motivation:

I am very influenced by the people around me. It affects me because I feel that I can lose trust, that I can lose face. (P5)

## 5. Discussion

This study aimed to gain a deeper understanding of the motivational forces driving nurse leaders toward developing a health-promoting work environment. The nurse leaders highlighted how their sense of inner responsibility, trusting relationships, mastery, and recognition served as motivational forces in developing a health-promoting work environment. Through the interpretation of the data, it emerged that leaders' motivations are intricately linked to their interactions with others and are deeply rooted in community engagement. Our findings show that the leaders' responsibility for others with an inner desire to promote everyday wellbeing shows that the leaders were driven by nonegoistic motives, which is in line with previous studies [15, 16, 38, 39, 44]. This aspect of motivation was also linked to the prosocial motivation previously examined by Grant and Berry [45]. Humans' desire to take on responsibilities involves being conscious of their ethos [46]. Leaders who are in touch with their ethos, metaphorically feeling “at home,” are internally driven, living in freedom and harmony with themselves, and maintaining integrity and authenticity in their actions [16, 34]. An authentic leader who is aware of his ethos can infuse meaning and inspiration into the work environment. When a leader's basic values harmonize with their actions, it not only fosters trust and positive emotions among employees but also cultivates a shared identification with the leader's vision. This, in turn, significantly enhances job satisfaction and engagement in the workplace [23, 47].

As in earlier studies, the findings in this study demonstrate that trusting relationships in the workplace are essential elements to enhance nurse leaders' self-confidence and independence in leadership [15, 38] leading to engagement and discovery of new and constructive perspectives for both thinking and action [5] and improving nurse leader's work-related wellbeing [48]. A recent systematic review explored the crucial role of trust in leadership, revealing that it is at the core of the leader-employee collaborative relationship. Trust was identified as a central driver of organizational success and was associated with expectations of behavior [49]. Furthermore, Okello and Gilson [50] found in their systematic review that workplace trust relationships influence the motivation of health workers. However, the three-dimensional aspects of trusting relationships, highlighted in this study, have not been widely explored in motivation studies in nursing leadership. Previous studies support the finding that relationships with superiors and employees are crucial for nurse leaders' wellbeing and motivation which in turn significantly contributes to creating and maintaining a healthy work environment [48, 51–53]. However, our findings indicate that trust is a prerequisite for a supportive relationship between the leader and both superiors and employees. Trust is a fundamental value in life and is an essential prerequisite for human interaction. It emerges as the other is receiving us. Exposed to one another in interdependence, trust comes forth [54].

Autonomy, which is an essential motivational factor [31, 32], is understood in the nursing leadership context as a way for superior leaders to show trust to nurse leaders. However, this study indicates that without adequate information, support, and training, autonomy alone cannot motivate leaders to develop a health-promoting work environment. Previous study also highlights the importance of a balance between nurse leaders having autonomy while maintaining a close relationship with their superiors [55, 56]. In other words, autonomy presupposes support, learning, and competence as critical job resources. This finding aligns with the job demands-resources model [57] where job resources emerge as a crucial factor for the work engagement of nurse leaders, promoting their motivation [3]. According to the findings in this study, learning is closely linked to a sense of mastery, an essential factor for nurses' self-efficacy and motivation for leadership [58]. This finding, in line with previous studies, further emphasizes the importance of leaders' empowerment, which is closely linked to learning and autonomy, for their job satisfaction, motivation, role satisfaction, and managerial self-efficacy [48, 59]. Learning serves as a driving force in human life, encompassing development, personal growth, and being in a continuous process between oneself and others in an attempt to become a whole person, a person one wants to be [60].

Moreover, the findings in this study underscore the crucial connection between an employee's sense of mastery and that of their leader as also noted by Kohnen et al. [3]. When leaders cultivate an environment that fosters mastery for their team members, it not only enhances the employees' motivation and commitment but also exerts an indirect positive influence on the leader's motivation and commitment, according to this study, learning and training encompass administrative and technical aspects and deal with various interpersonal and ethical challenges that arise in everyday work, which is congruent with previous studies [38, 61, 62]. A recent study further supports this finding and highlights that mentoring programs for leaders should not only guide them in addressing managerial and administrative challenges but also support them in handling stressful ethical situations [63].

This study emphasized that recognizing nurse leaders by superior leaders is crucial to nurturing their motivation and commitment. The recognition of leaders means that leaders as unique human beings with unique skills, competencies, qualities, and potential are respected by their superiors [60]. The lack of recognition of nurse leaders can lead to a sense of loneliness [55]. The active involvement of nurse leaders in the decision-making process can enhance their sense of recognition, as seen in previous studies [58, 64, 65]. According to previous studies, leaders' lack of involvement in decision-making has negative consequences for nursing care, including high turnover, heavy workloads, professional burnout, and reduced quality of health services [65]. The findings in this study highlighted that when leaders experience a lack of recognition or feel that their voices are not heard by higher-level leadership, it can lead to frustration. This frustration can lead to a feeling of working against their values, which might lead to moral distress as seen in previous studies [16, 66, 67].

## 6. Strengths and Limitations

The strength of the study lies in its adherence to Lincoln and Guba's criteria [68] to ensure trustworthiness and the Standards for Reporting Qualitative Research [69] to enhance transparency (Supporting File 1). This study was conducted by a balanced research team comprising both male and female members, each with expertise in qualitative research methods spanning nursing and psychology. In addition, while this study primarily adopts a caring science perspective, the findings have also been discussed from a psychological viewpoint. This approach, known as researchers and theory triangulation, is considered a notable strength [70, 71].

A potential limitation in hermeneutic studies is the researchers' insufficient awareness of their preunderstanding [41]. To address this limitation and ensure transparency, credibility, and confirmability, the researchers actively participated in all phases of the study. Feedback and comments were utilized to check the quality and credibility of the study. In addition, direct quotes from participants and the use of an interview guide were employed to maintain a clear focus during all interviews. Our preunderstanding is reflected in the introduction through the presentation of previous studies and theoretical perspectives.

Furthermore, due to geographical distances and challenges in meeting physically, some interviews were conducted via the digital platform Teams. Utilizing team video communication presents both strengths and limitations. Norway's elongated shape, dispersed settlements, and substantial distance from major cities often exclude nurse leaders' voices from distant districts in various research projects. The use of digital platforms can help mitigate this limitation. However, in face-to-face interviews, facial expressions and body language facilitate better communication, enabling researchers to gather additional data through observation, which is an important source of rich data [72, 73].

Another possible limitation of the study is that of the 13 participants, only 2 were male. This gender imbalance could potentially impact the study's results and conclusions. Despite the limitations, however, the interviews provided rich data, and the participation of 13 leaders from different contexts may have strengthened the transferability of the study to other contexts.

## 7. Conclusion

The nurse leaders emphasized how their sense of inner responsibility, trusting relationships, mastery, and recognition served as motivational forces in developing a health-promoting work environment. These motivational forces lead nurse leaders to be in touch with their ethos, which means a sense of “at-homeness” that encompasses authenticity and an experience of being rooted in one's core values. However, the motivational forces and values often remain implicit and overlooked. This study highlights the importance of raising awareness through reflection and articulating nurse leaders' underlying motives and values. Doing so transforms abstract concepts into tangible factors that stakeholders can grasp. In particular, this insight provides valuable guidance to politicians and decision-makers on facilitating improved working conditions for nurse leaders, thereby maintaining and enhancing their motivation.

## 8. Implications for Nursing Leadership

This study highlights the importance of superior leaders and policymakers focusing on promoting nurse leaders' autonomy and providing opportunities that allow them the freedom to make decisions and take action. In addition, education, training, and mentoring programs addressing administrative, professional, and ethical challenges are crucial for enhancing leaders' motivation, which is a key element in developing a health-promoting work environment. Furthermore, fostering leadership networks where nurse leaders can exchange experiences and address daily challenges is essential. Involving nurse leaders early in decision-making and valuing their input ensures their expertise shapes policies and practices, which in turn motivates them to take ownership in fostering healthier workplaces. Since human motives and values are expressed through actions and interactions, further research should focus on the actions that nursing leaders employ to develop a health-promoting work environment.

## Figures and Tables

**Table 1:** An overview of the characteristics of participants and contexts.

Age	Gender	Additional education	Leadership experience	Context
32–57 Mean: 42,9	Male: 2 Female: 11	Leadership: 9 Intensive Care Nursing: 1 Elderly Care and Geriatrics: 1 None: 2	0.5–14 years Mean: 7,2	Nursing homes: 5 Home care: 1 Medical Department: 3 Surgical Department: 2 Intensive Care Unit: 1 Physical Medicine and Rehabilitation: 1

**Table 2:** Description of Fleming's four phases [41].

Phase 1	Reading all interview texts to gain a holistic understanding that reflects the fundamental meaning of the text as a whole
Phase 2	Examining sections and sentences to reveal meaningful units
Phase 3	Relating the sections and sentences to the meaning of the whole text
Phase 4	Identifying sections that appear to be representative of the shared understanding between the researcher and participants

## Data Availability

The data that support the findings of this study are available from the corresponding author upon reasonable request.

## Supporting Information

See Supporting File 1 for detailed reporting criteria used in this study. The Standards for Reporting Qualitative Research (SRQR) consist of 21 items aimed at improving the transparency of qualitative research by providing clear standards for reporting.
